# Conditions for CNT – Coated Textile Sensors Applied to Wearable Platforms to Monitor Limb Joint Motion

**DOI:** 10.1007/s10916-021-01709-8

**Published:** 2021-02-19

**Authors:** Da-Hye Kang, Joo-Hyeon Lee, Jeong-Whan Lee, Hyun-Seung Cho, Seon-Hyung Park, Kang-Hwi Lee, Seung-Jin Kang

**Affiliations:** 1 Department of Clothing & Textiles, College of Human Ecology, Yonsei University, 50 Yonsei-ro, Seodaemun-gu, Seoul, 03722 Republic of Korea; 2 Department of Biomedical Engineering, College of Biomedical &Health Science, Konkuk University, 268 Chungwon-daero, Chungju-si, Chungcheongbuk-do 27478 Republic of Korea

**Keywords:** Carbon nanotube textile sensor, Limb motion sensor, CNT, Textile strain gauge, Wearable sensor

## Abstract

Despite recent research on joint motion measurement to monitor human body movement, current measurement techniques and tools have significant limitations, including requiring large space for measurement and causing discomfort in test subjects wearing motion sensors. Our study aims, first, to develop carbon nanotube (CNT)-based textile joint motion sensors. Second, ours study aims to identify the most suitable CNT-based sensor structure and attachment method for use on a wearable platform during general exercise speeds. Lastly, we used these sensors on the human body, using sleeves and legs to find the most stable location, and we used the CNT-based sensor condition to monitor joint motions. We utilized our CNT-based sensor, which has proper elasticity as well as conductivity, and applied it to the elbow and knee joints. Based on the strain gauge principle, we monitored the variance of electric resistance that occurred when the CNT-based sensor was stretched due to limb motion. Our study tested 48 types of sensors. These sensors were applied to the CNT using different base knit textiles as well as different attachment methods, layers, sensor lengths, and sensor widths. The four most successful sensor types, which showed superior efficacy over the others in joint motion measurement, were selected for further study. These four sensors were then used to measure the elbow and knee joint motions of human subjects by placing them on different locations on sleeves and legs. The CNT knit textile sensors best suited to measuring joint motions are those with a double-layered CNT knit and 5 cm long × 0.5 cm or 1 cm wide sensors attached to a polyester¬-based knit using a welding method. The best position for the sensor to more stably monitor joint motions was the “below hinge position” from the elbow or knee hinge joint. Our study suggests an alternative strategy for joint-motion measurement that could contribute to the development of more comfortable and human-friendly methods of human limb motion measurement.

## Introduction

During recent years, efforts have been made to study joint-motion measurement as a way to monitor body movement [1–3]. This is based on the recent anticipated demand for devices that analyze body movement in the field of educative sports and rehabilitation. Human body motions are monitored using wearable strain sensors that have been developed based on various sensing mechanisms including resistive, capacitive, or piezoelectric sensing [4–7]. These wearable strain sensors made of materials including carbon nanotube (CNT), silicone, or ZnO nanostructures are applied to detect movements of body including elbow, finger, hand, knee, face, and chest [8]. However, the preexisting techniques to measure body movement have numerous limitations. For instance, 3-dimensional motion capture systems using infrared rays are limited in their ability to measure detailed joint movements or to monitor the hinge joints of the body in real life. In addition, these systems are expensive, and the technical equipment required for them to work usually requires a large amount of space. Xsens, for instance, has developed an MVN motion-capture suit with 17 inertial trackers [9]. Although the suit measures and transmits joint angle data using a wireless connection to a computer to determine body posture, the rigid sensors attached to the suit are reportedly hard and uncomfortable to wear. Danfoss, on the other hand, has developed a wireless flexible sensor called “PolyPower” that senses body joint movement [10]. Stretchsense has created a lightweight, flexible, fabric stretch sensor to measure sports motions [11]. It is composed of sensors and circuits and can be sewed into sports suits, gloves, and other smart apparel to test body movement. Heddoko developed a smart garment which, by measuring limb movements using a wearable sensor, can simultaneously scan the movement and prevent worker injury [12]. Although numerous methods to measure motions have been studied, and fabric motion sensor is still under development, in order to more accurately and comfortably measure human limb movement.

Our study explores an alternative approach to monitor joint motion using a wearable platform. This approach includes the development of CNT-coated textile sensors to monitor joint motion in the human body based on the strain gauge principle. We designed 48 types of CNT-coated textile motion sensors with different structures and applied them to the elbow hinge joint of a dummy arm. Among the 48 types, we identified the types of CNT knit sensors that are more suitable for measuring and gathering data during limb movements. Lastly, we ascertained which sensor locations on the sleeves and legs lead to the most accurate and motion capture results.

## Methods

### Principle of measurement

Changes in electric resistance depending on joint motion were measured using the CNT-coated textile strain gauge sensors. The measurement used in this study was based on the strain gauge principle, which states that the area and length of a sensor changes when a strain gauge is placed under compressive force within the elastic limit. The change in resistance is measured by the equation shown in (eq. 1) [13, 14].

1 $$ R\left(\varOmega \right)=\rho L/A $$

R: resistance (ohm); ρ: resistivity (ohm∙meter); L: sensor length (meters); A: cross-section area of sensor (meter squared).

### Implementation of the textile motion sensor

CNT, which has excellent mechanical and electrical properties, was used as a sensor material for this study. To implement the highly sensitive strain sensors, we needed to adopt a material with proper conductivity as well as stretchability. Single-wall carbon nanotube (SWCNT) was applied as the main material to impose conductivity to the textile sensor, while urethane, a sort of elastomer, was used to impart stretchability.

**Fig. 1:**
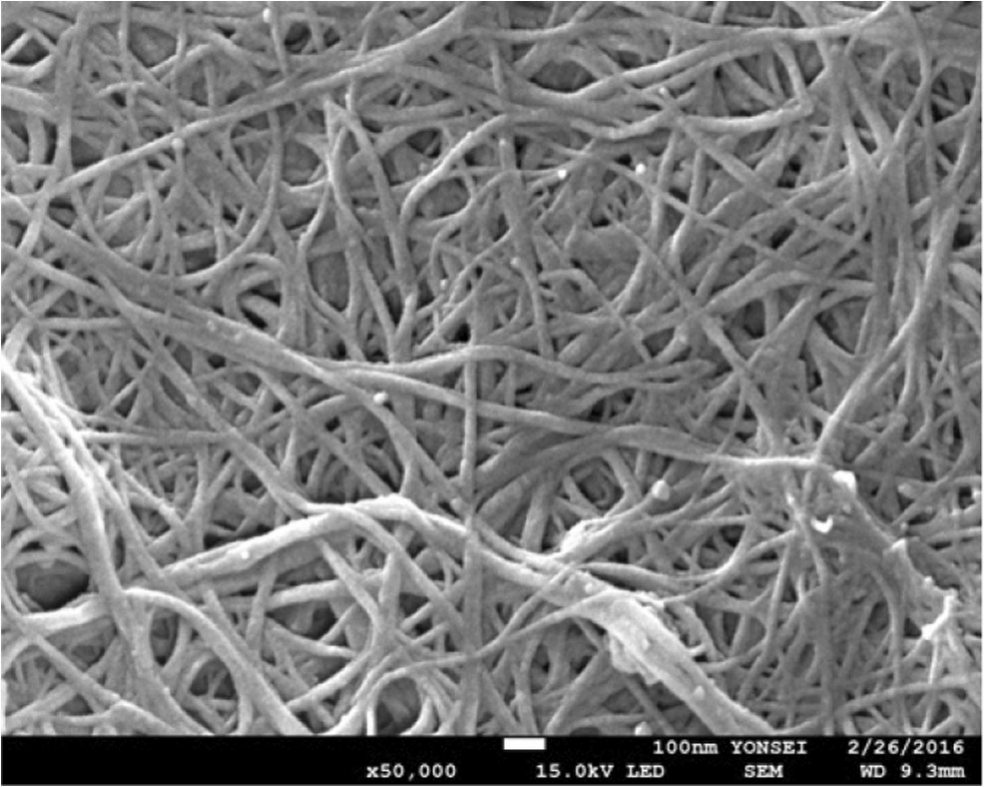
Scanning electron microscope (SEM) image of the single wall carbon nanotube coated knit textile (×50,000)

First, we made a conductive and stretchable slurry and used it to coat the base textile layer. This conductive slurry is mainly composed of single-walled carbon nano black powder, water, and urethane. To prepare the single-walled carbon nano black slurry, pure water with a 100% weight ratio and carbon nano black powder with a 60% weight ratio were dispersed in a tank and stirred. A urethane emulsion was added to the dispersed slurry at a weight ratio of 25% and further dispersed once more into the continuous-type bead mill. A highly elastic jersey knit textile (77% polyester / 23% polyurethane) selected on the basis of the result from the pilot study on its growth and recovery property after repeated stretch, was added to the single-walled carbon nano black slurry as a base substrate. The knit textile was put in a desiccator in a vacuum state so that the single-walled carbon nano black slurry sufficiently deposited and coated it. The deposited textile was placed in a dry curing machine (max. 200 °C) and cured at 120 °C for 1 h to complete the preparation for a stretchable and conductive textile as the material of the textile motion sensor (Fig. 1).

**Fig. 2:**
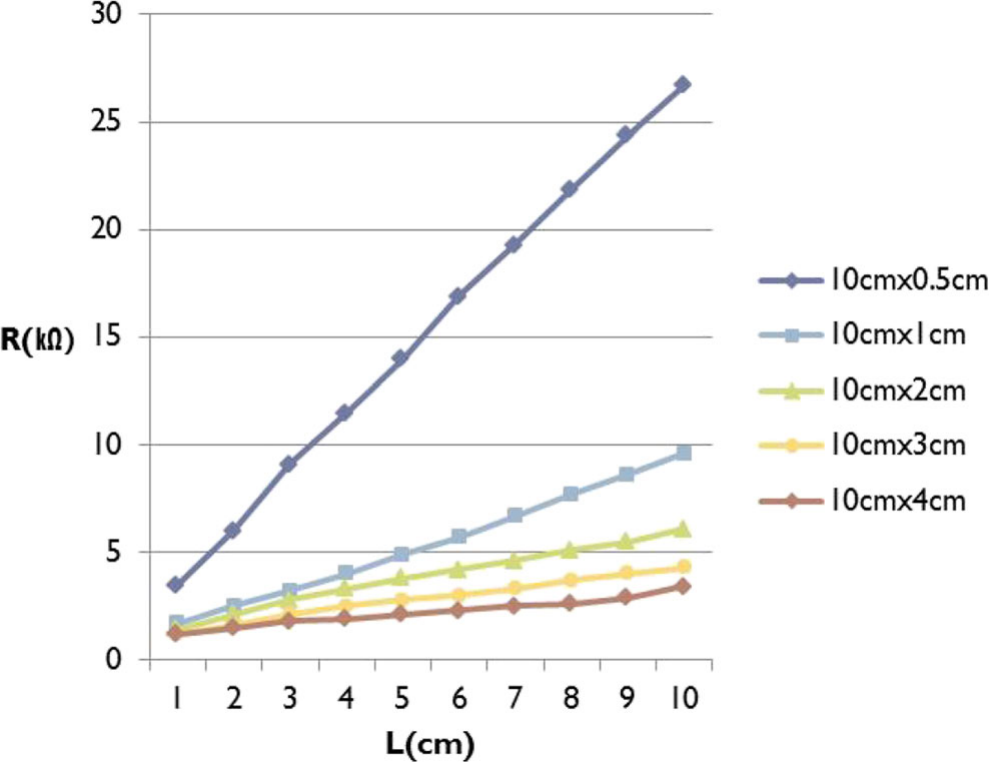
Linear relationship between length of CNT-coated knit textile and electric resistance

To test whether our CNT-coated knit textile is suitable for use as a sensor material, the consistency of electric resistance change in response to changes in the length of the CNT-coated knit textile (from 1 cm to 10 cm) was measured by 1 cm interval using 5 different CNT-coated knit textiles (10 cm × 0.5 cm / 10 cm × 1 cm / 10 cm × 2 cm / 10 cm × 3 cm / 10 cm × 4 cm). The length of CNT-coated knit was set at 10 cm, since 3-dimensional motion capture system showed the most elastic point 5 cm above and below the joints [15]. As shown in Fig. 2, a proportional linear relation was observed between electric resistance and length of the CNT-coated knit textiles with different width. Narrower textile had higher electric resistance. Thus, CNT-coated knit textile of 0.5 cm or 1 cm width was determined to be appropriate for use as a motion sensor material based on the strain gauge principle.

**Fig. 3:**
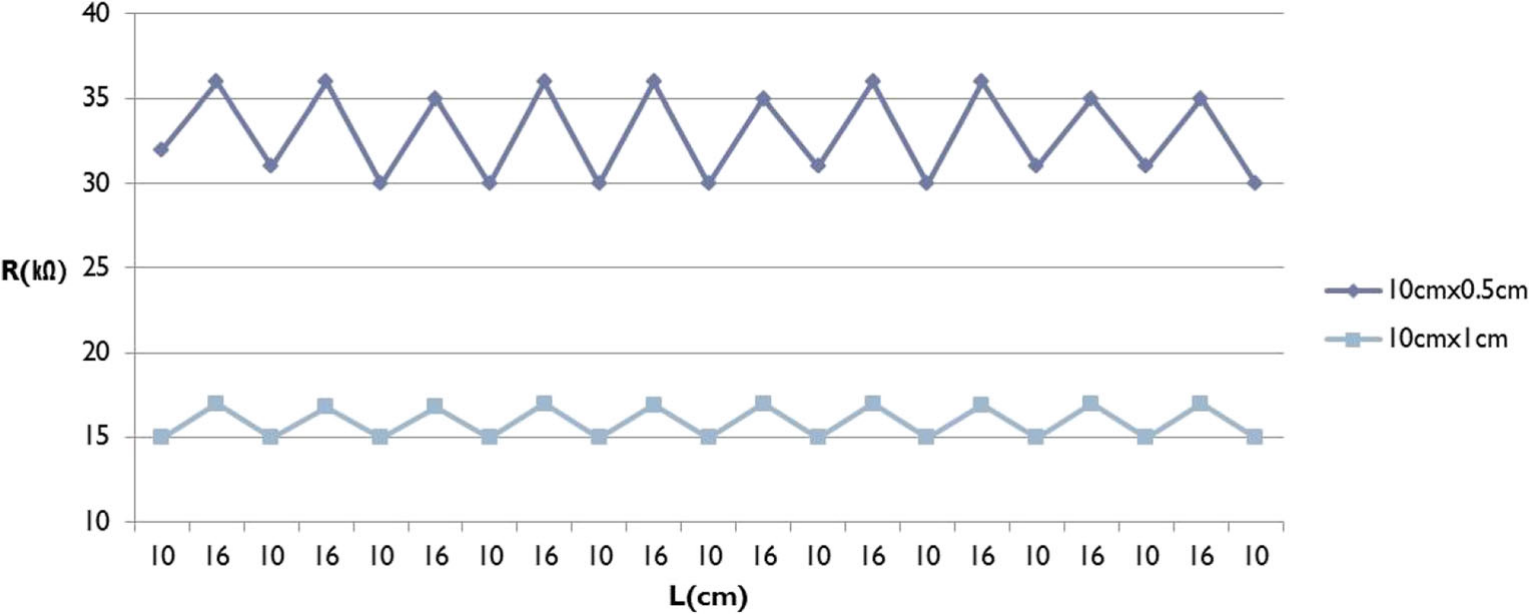
Stability of change in electric resistance extension and contraction of CNT-coated knit textile

In next step, the stability of change in electric resistance upon repeated extension and contraction of the CNT-coated knit textile was evaluated in order to ascertain whether the textile is adequate as a sensor within the range of limb joint motion. The experiment was designed based on strain gauge theory, which predicts that when the CNT-coated knit textile is stretched, its length should increase and its cross-sectional area should decrease, resulting in an increase in resistance. For this, a CNT-coated knit textile of 10 cm length and 0.5 cm or 1 cm width was used as determined in Fig. 2. 10 replicated actions were measured in which the CNT coated knit textile was extended to 16 cm and then contracted back to 10 cm. The extension length, 16 cm, was determined based on evidence that the maximum extension rate of skin in some sports motions is 60% [16, 17]. As shown in Fig. 3, there was a stable change in electric resistance upon extension and contraction of the 10 cm × 1 cm CNT-coated knit textile, whereas 10 cm × 0.5 cm textile showed less stable but more pronounced change in electric resistance. Based on these preliminary evaluations, we confirmed that our CNT-coated knit textile is an adequate sensor material for limb motion sensing. Following this validation procedure, a CNT-coated knit textile sensor (abbreviated as “CNT sensor’) of 0.5 cm or 1 cm width was devised for use in this study.

### Resistance measurement

Data acquisition for resistance measurement during joint movements was made using Graphtec GL7000. In brief, wristlets were vertically installed with various CNT sensors and applied to a dummy arm, right sleeve of tops, or right knee of pants. In case of wristlets applied to a dummy arm, contraction and relaxation of the elbow joint were made using dynamometer (CON-TREX MJ). Three replicates of 10 motion sets were measured with 60 s interval between each set. The speed of one motion was set as 0.5 Hz, a general moderate exercising speed. Two snap buttons were attached at both ends of the sensor on the wristlets, which were connected to the oscilloscope, Graphtec GL7000. The voltages measured were saved and converted to resistance data form using GL-Connection software. Temperature and humidity of the laboratory were maintained at 25 °C and 55 ± 5%, respectively, during the experiment.

## Experiments

### Experiment 1: Evaluation of CNT sensor conditions

#### Experimental objectives

The objective of this experiment is to identify the most efficient form of sensor and attachment method to sense joint motion by testing the CNT sensor under diverse conditions.

#### The CNT sensor attached to wristlets

48 types of CNT knit were prepared to implement the most efficient CNT sensor to sense joint motion. Variables considered for CNT sensor generation include the form of the sensor, the type of base knit textile, and the attachment method.

The formal properties of the sensor included the number of CNT layers and the length and width of the sensor (as determined by the strain gauge principle). Two kinds of highly elastic knit popularly used for sportswear were adopted as the base textile for the sensor. These two base textiles with most outstanding flexibility were selected among 10 commonly used elastic sportswear materials through a resilience test. One was made with 74% nylon and 26% polyurethane had the label “L” (L-knit), while the other was made with 77% polyester and 23% polyurethane had the label “W” (W-knit); both were coated with CNT. These fiber compositions which include higher rates of elastomer, polyurethane with polyester or nylon are popularly used in various forms of sportswear.

**Fig. 4:**
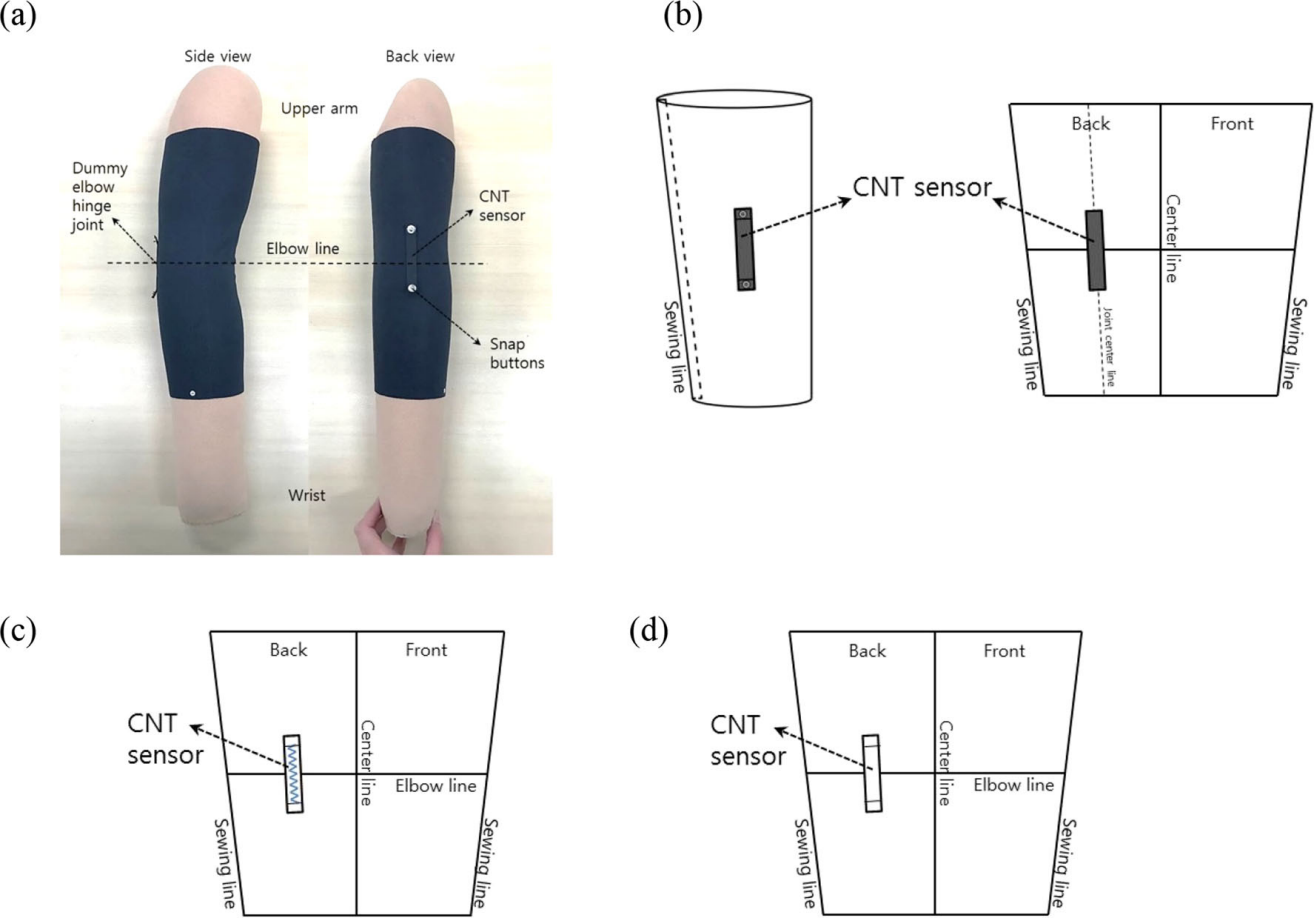
Wristlet fabrication. (**a**) Wristlet with CNT sensor worn on dummy arm; (**b**) schematic diagram of wristlet; (**c**) schematic diagram of wristlet with sensor attached using sewing method; (**d**) schematic diagram of wristlet with sensor attached using welding method

The wristlets were generated based on the dummy arm size (see section 3.1.3.) to be installed with the CNT sensor. On each wristlet’s surface, each CNT sensor was vertically distributed along the joint center line (Fig. 4a, b). Snap buttons were attached at both sensor ends as interconnection parts to measure electricity.

Two attachment methods were used to attach the sensors to the surface of each wristlet. These methods include the sewing method, where the sensor was attached via a zigzag stitch using sewing machine (Fig. 4 (c)), and the welding method, where the sensor was attached via an adhesive film (Fig. 4 (d)). The film used for the welding method, which is popularly applied to apparel production, was a non-sewing double-side adhesive with excellent elasticity and recovery rate, and it was applied using a heat press machine. Sensor layers were composed of either single- or double-layers. Sensors with three different lengths—5 cm, 10 cm, and 15 cm—and two different widths—0.5 cm and 1 cm—were generated.

Combining all of the variables (2x2x2x3x2) reveals that 48 types of sensors were devised, featuring two types of base knit textile (L-knit, W-knit), two attachment methods (sewing, welding), two numbers of layers (single- and double-layers), three different lengths (5, 10, 15 cm), and two different widths (0.5, 1 cm).

#### Experimental setup

**Fig. 5:**
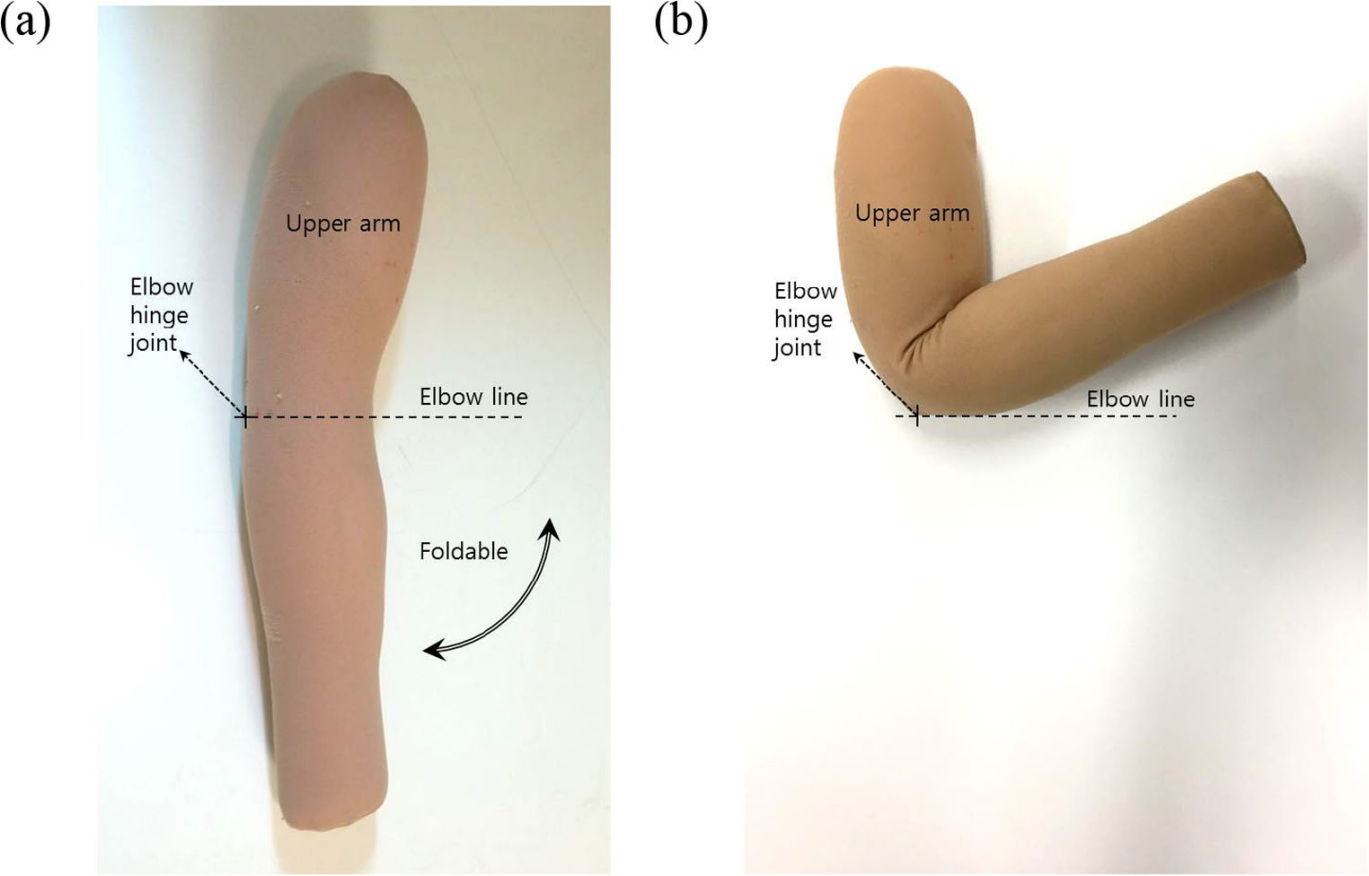
A dummy arm with a foldable elbow joint. (**a**) Uncontracted state; (**b**) contracted state

A dummy arm with a foldable elbow hinge joint was made based on the standard medium arm size of Korean males and was used for Experiment 1 (Fig. 5).

**Fig. 6:**
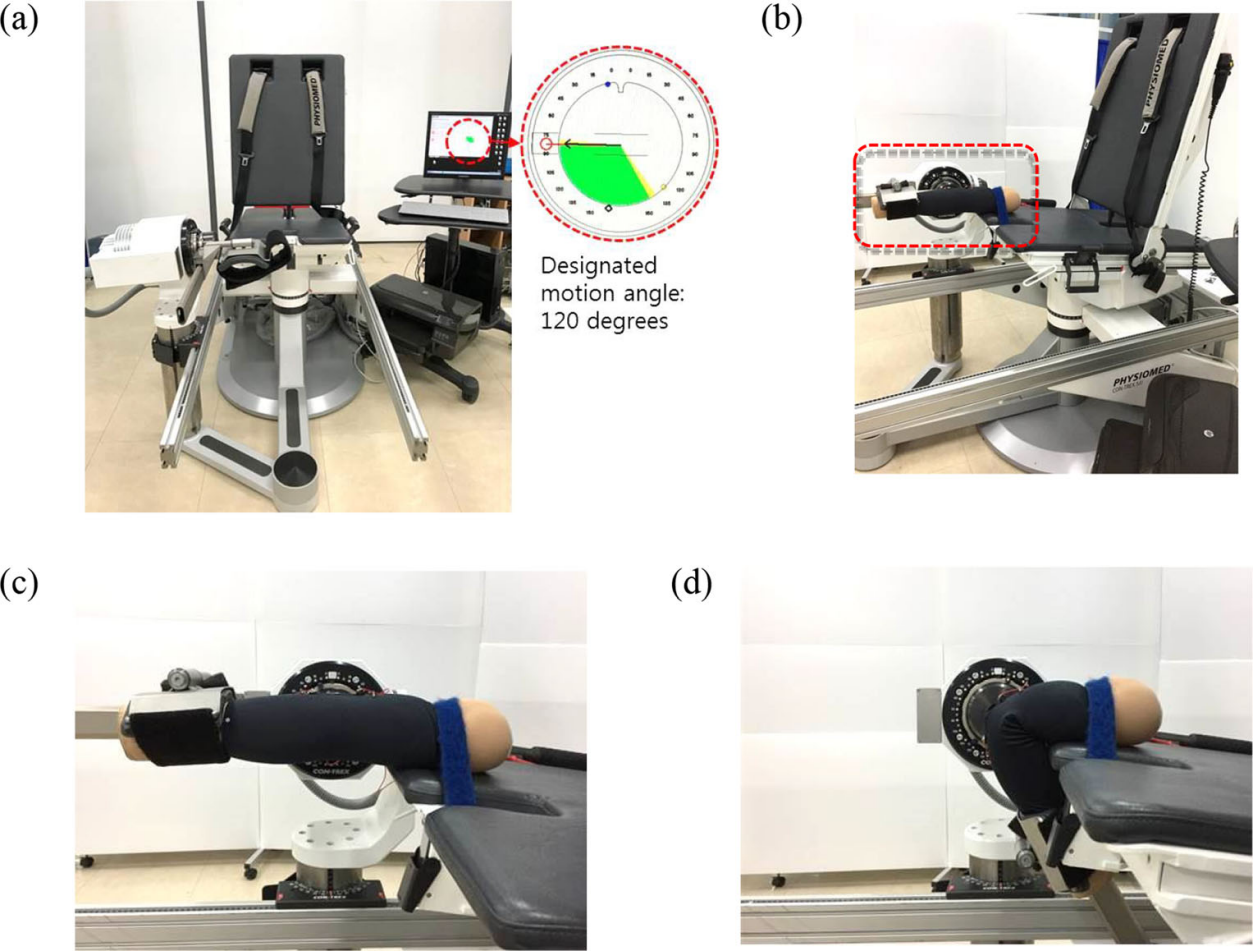
Condition test of a CNT sensor attached to a fixed dummy arm. (**a**) Overview of the experimental setting; (**b**) experimental setting equipped with dummy; (**c**) relaxed state; (**d**) 120° contracted state

Wristlets with CNT sensors were attached to a dummy to evaluate the efficiency of the CNT sensors condition. Top and bottom parts of the wristlet were immobilized to dummy arm with sewing pins to minimize slipperiness. Starting with the uncontracted dummy arm, one complete contraction and relaxation of the elbow joint was considered a single motion unit. One motion set was composed of 10 motion units, and all motion sets were spaced out 60 s apart. Three replications of motion sets were measured for electric resistance changes using an oscilloscope. The speed of one motion unit was set to 0.5 Hz, and the maximal angle of the contracted elbow joint was restricted to 120 degrees [18] using a dynamometer (CON-TREX MJ) (Fig. 6).

#### Experimental results

Three distinct aspects were evaluated based on electric resistance changes found during condition tests of 48 types of CNT sensors. These include (1) the degree of appearance of double peaks in signal morphology, (2) the consistency of baseline across the three motion sets, and (3) the consistency in measurement across the three motion sets. The sensors that did not satisfactorily meet these criteria were ruled out as followings, and the remaining four types were selected as suitable for the measurement of joint motion.

Dramatic double peaks were observed in sensors attached using the sewing method (Table 1 (a), (b), (c), (d)), whereas reduced or absent double peaks were observed in sensors attached by the welding method (Table 1 (e), (f)). Among the sensors attached by the welding method, double peaks were observed in longer sensors of 10 cm or 15 cm (Table 1 (a), (b), (c), (d), (e), (f)). Meanwhile, the shortest sensors with (5 cm length) exhibited a more consistent baseline (Table 1 (g), (h), (i), (j)). Moreover, in the sensors that were 5 cm in length, three sets of repeated measurements resulted in more stable and consistent changes in resistance without any decline of baseline (Table 1 (g), (h), (i), (j)). These results indicate that attachment method and sensor length are the critical variables influencing baseline fluctuation and the appearance of double peak phenomena. With this observation in mind, we ruled out sensors attached using the sewing method as well as those of longer lengths (10 cm or 15 cm).

**Table 1:**
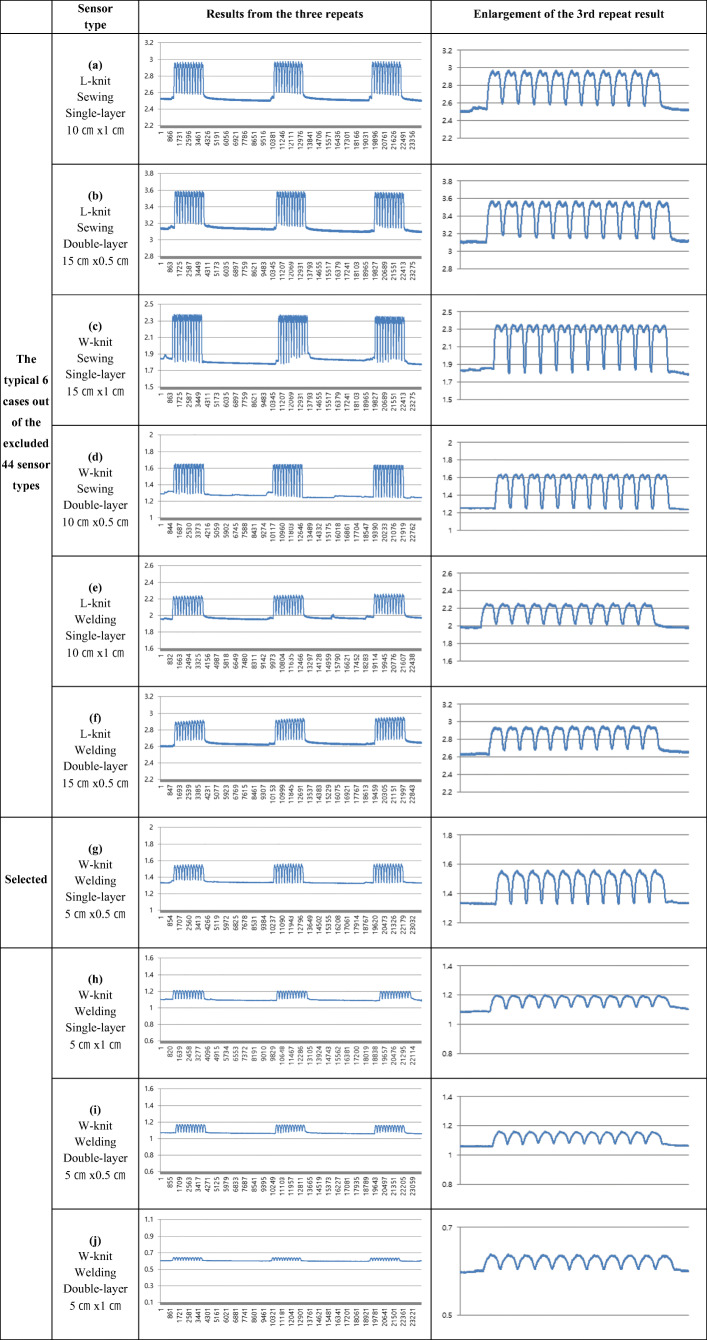
Electric resistance change in motion depending on the research variables. (Y axis: resistance (Ω); X axis: repeats of the motion)

Among the remaining sensor types, there was a greater tendency for baseline fluctuation across the motion sets in the nylon-based L-knit sensors (Table 1 (e), (f)), but not in most of the W-knit sensors (Table 1 (g), (h), (i), (j)). With regard to the number of sensor layers, both single- and double-layer sensors showed a similar tendency with regard to baseline change, although double-layer sensors were slightly more stable and gave more consistent results than single-layer sensors (Table 2). For sensor width, the 1 cm width sensors were slightly more stable and consistent (Table 1 (h), (j)) than the 0.5 cm width sensors (Table 1 (g), (i)), although both widths showed similar patterns of measurement overall (Table 2).

**Table 2:** Average measurement of baseline change across the 3 motion sets (Ω)

Base textile/Attach- ment method Form factor	**L-knit** **sewing**	**L-knit** **welding**	**W-knit** **sewing**	**W-knit** **welding**
**Single-layer 5 × 0.5**	0.001	0.004	0.003	0.001
**Single-layer 5 × 1**	0.002	0.003	0.000	0.003
**Single-layer 10 × 0.5**	0.002	0.006	0.008	0.011
**Single-layer 10 × 1**	0.002	0.004	0.006	0.001
**Single-layer 15 × 0.5**	0.003	0.003	0.003	0.004
**Single-layer 15 × 1**	0.006	0.005	0.003	0.001
**Double-layer 5 × 0.5**	0.003	0.002	0.001	0.002
**Double-layer 5 × 1**	0.002	0.001	0.002	0.001
**Double-layer 10 × 0.5**	0.005	0.004	0.000	0.001
**Double-layer 10 × 1**	0.004	0.001	0.000	0.000
**Double-layer 15 × 0.5**	0.003	0.006	0.005	0.002
**Double-layer 15 × 1**	0.005	0.002	0.002	0.001

(*Light-shaded: baseline lift resistance change after motion ≤0.005 Ω; middle-shaded: baseline lift resistance change after motion ≤0.002 Ω; dark-shaded: baseline lift resistance change after motion <0.001 Ω)

Following these results, all of the sensors that were made of nylon-based L-knit, that were attached using the sewing method, and that were 10 cm or 15 cm in length were excluded. A total of four types of CNT sensors—those with a W-knit, 5 cm in length, and attached by the welding method—were ultimately selected for further analysis. These included single-layered 5 cm × 0.5 cm/5 cm × 1 cm sensors and double-layered 5 cm × 0.5 cm/5 cm × 1 cm sensors (Table 1 (g), (h), (i), (j)).

### Experiment 2: CNT sensor attachment location test

#### Experimental objectives

The experiment objective is to identify the attachment locations on the sleeves and legs that are most efficient for measuring resistance changes in joint motions. We used the four sensor types selected in experiment 1 (single-layered 5 cm × 0.5 cm/5 cm × 1 cm, and double-layered 5 cm × 0.5 cm/5 cm × 1 cm). Experiments 2-A and 2-B were performed in order to determine the most effective attachment positions for the CNT sensor on the elbow joint and knee joint, respectively.

#### Human subjects

Five males consented to participate as subjects for the study. The physical condition of each subject is listed in Table 3.

**Table 3:** Physical condition of subjects

**Subject**	**Gender**	**Age (yrs)**	**Height (cm)**	**Weight (kg)**	**Elbow circumference (cm)**	**Knee circumference** **(cm)**
1	Male	33	187	84	28	40
2	Male	34	173	73	25.5	36
3	Male	33	180	78	26.5	39
4	Male	35	178	61	26	36
5	Male	34	187	98	29.5	42

The difference between actual human joint motions and dummy arm joint motions were taken into consideration when equipping the wristlets with the CNT sensors. With human subjects, CNT sensors can stretch to great extremes or extend inconsistently, since human body joints can be pointy or irregular according to the subject’s somatotype. Therefore, in performing Experiment 2, which used human subjects, the sensor mounting position location around the joint area on the wearable platforms was varied. In case of Experiment 2, although sensors were mounted exactly 1 cm below or 1 cm above the hinge joints of the elbow and knee, when each subject moved the distance between hinge joints and sensors were inconsistently extended according to each subject’s body type.

#### Experiment 2-a

##### The CNT sensor and wearable platform

**Fig. 7:**
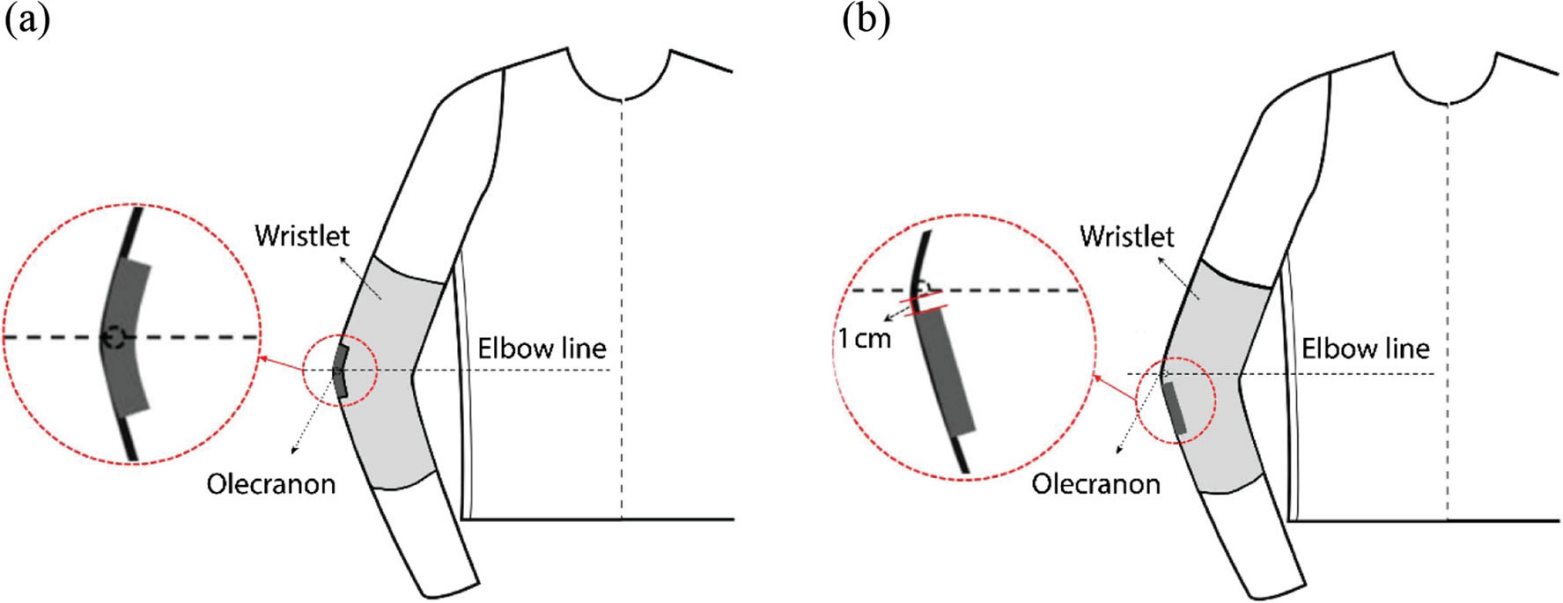
Schematic diagram of CNT sensor location attached around the olecranon. (**a**) Joint-hinge position; (**b**) below-hinge position

Two locations on the elbow joint were used for the four types of CNT-sensor attachment. The center of the sensor was attached either to the olecranon (the elbow hinge joint), called the “joint-hinge position,” or below the olecranon, called the “below-hinge position.” The “below-hinge position” was determined to be 1 cm under the actual olecranon of each subject (Fig. 7). The upper end of the sensor was placed 1 cm below the olecranon in order to stably fix the interconnection part located at the end of the sensor.

In the matter of experimental design, a total of eight sensor conditions (=4 × 2) on the wearable platform were set using four different sensor types (research variable #1) and two distinct attachment locations (joint-hinge and below-hinge position; research variable #2) around the elbow on a sleeve.

**Fig. 8:**
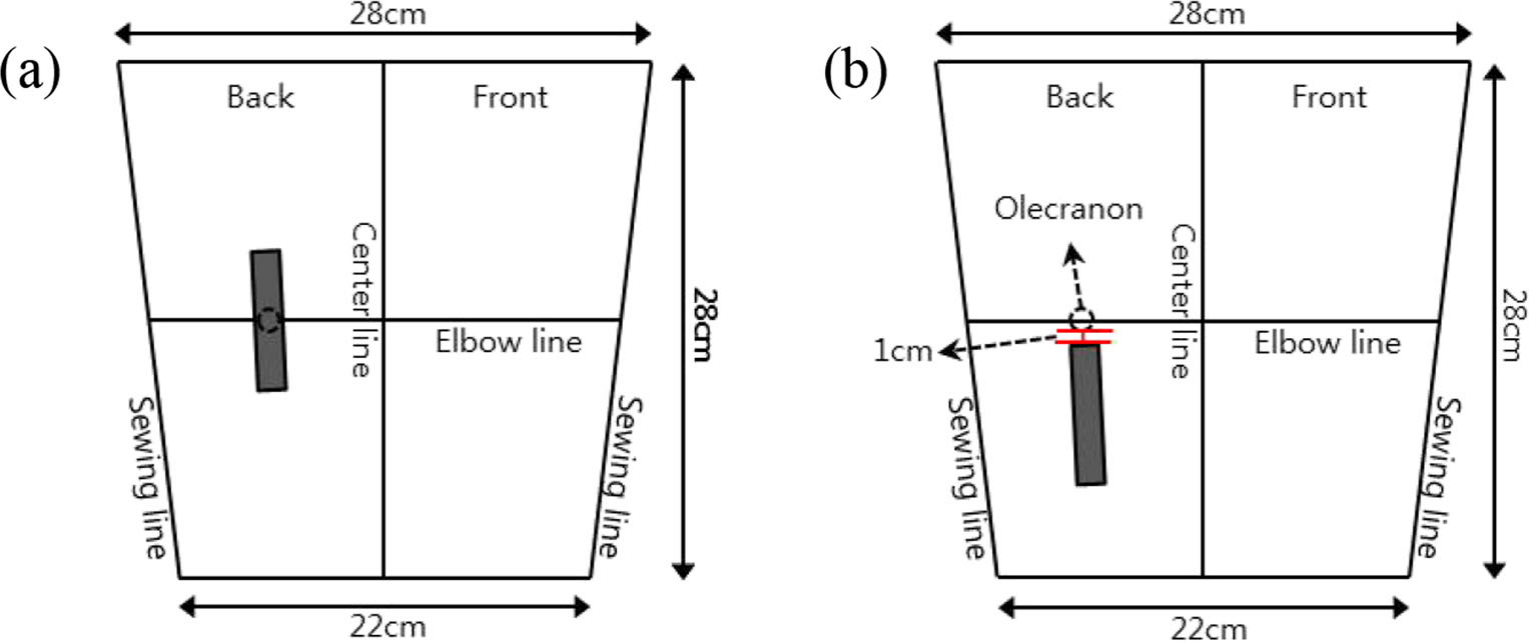
Schematic diagram of the CNT sensor locations on a wristlet. (**a**) Joint-hinge position; (**b**) below-hinge position

CNT sensors were located vertically around the olecranon position transferred to a wristlet. The elbow wristlets, a wearable platform, were made based on the medium size of upper arm circumference of Korean male to perform the CNT sensor location test (Fig. 8). All the materials for the fabrication of the wristlet were the same as in Experiment 1.

##### Experimental setup

**Fig. 9:**
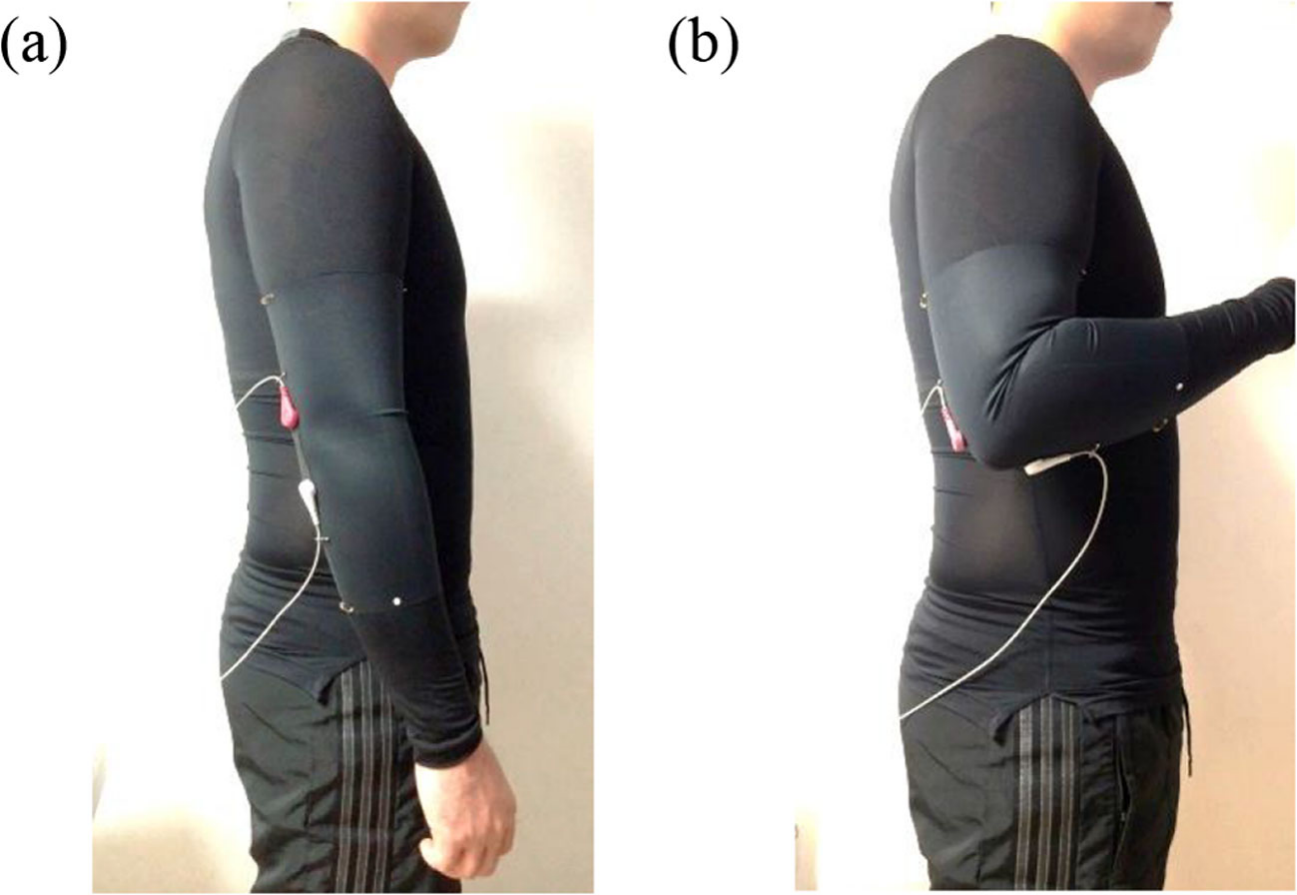
Test of elbow joint CNT sensor location in human subjects. Images of (**a**) relaxation of the elbow joint and (**b**) flexion of the elbow joint

Each individual subject wore a stretchy sports top, on the right sleeve of which a CNT-sensor attached wristlet was affixed. Both ends of the wristlet were immobilized to the sports top by sewing to minimize slipperiness. The subjects began with an uncontracted arm in an upright position. Complete flexion and relaxation of the elbow joint was considered one motion (Fig. 9). Each case was composed of 10 repetitive motions, and electric resistance changes were measured using an oscilloscope. The speed of one motion was set using a metronome as 0.5 Hz, which is widely considered to be a moderate exercising speed. The motion angle, which could vary depending on the subject’s arm thickness, was determined by having individuals bend forward forearm as far as possible and then stretch their arms (around 110°).

##### Experimental results

Electric resistance changes were measured while subjects performed joint motions wearing elbow wristlets equipped with four types of CNT sensors. Two distinct features were evaluated with regard to the characteristics of electric resistance changes in response to the motions. These include the baseline stability level and the presence of double-peak phenomena. Since Experiment 2 was performed on human subjects, the evaluation standards, such as the consistency of measurement during the repeats considered for Experiment 1, could not be comparably examined, due to the natural discrepancies in motion intensity between the individual human subjects.

Table 4 represents the average resistance change obtained from five human subjects performing ten repetitive elbow joint motions. The first row in Table 4 shows the average resistance when the subjects’ arms were relaxed, and the second row displays the peak values achieved when the subjects’ arms were flexed. The values in the third row represent the average resistance when the subjects’ arms were relaxed again after one motion unit of relaxation and flexion. The values in the final row represent differences in resistance during the relaxed state as measured before and after the motion. Values in the shaded boxes indicate when the averaged baseline lift after the motion was less than 0.005 Ω, implying that the baseline was stably maintained across repeats. In single-layered sensors, a baseline lift of more than 0.005 Ω was observed in the joint-hinge position, while the lift effect was decreased in the below-hinge position. In double-layered sensors, the baseline lift was slightly higher than 0.005 Ω in the joint-hinge position, whereas the below-hinge position showed more consistent and stable readings with minimal or no baseline lift (Tables 4 and 5).

**Table 4:** Repetitive measurement averages in elbow joint motion (Ω)

Form factor Position Motion	1 layer 5cm×0.5cm Joint	1 layer 5cm×0.5cm Below	1 layer 5cm×1cm Joint	1 layer 5cm×1cm Below	2 layers 5cm×0.5cm Joint	2 layers 5cm×0.5cm Below	2 layers 5cm×1cm Joint	2 layers 5cm×1cm Below
Relaxed	1.360	1.344	1.262	1.265	1.011	1.000	0.896	0.864
Flexed	1.559	1.549	1.409	1.373	1.103	1.091	1.019	0.975
Relaxed	1.368	1.349	1.268	1.268	1.015	1.002	0.894	0.864
Average baseline lift	0.008	0.005	0.006	0.003	0.004	0.002	−0.002	0.000

(Shaded: Baseline lift resistance change after one unit motion ≤0.005 Ω; joint: joint-hinge position; below: below-hinge position)

**Table 5:**
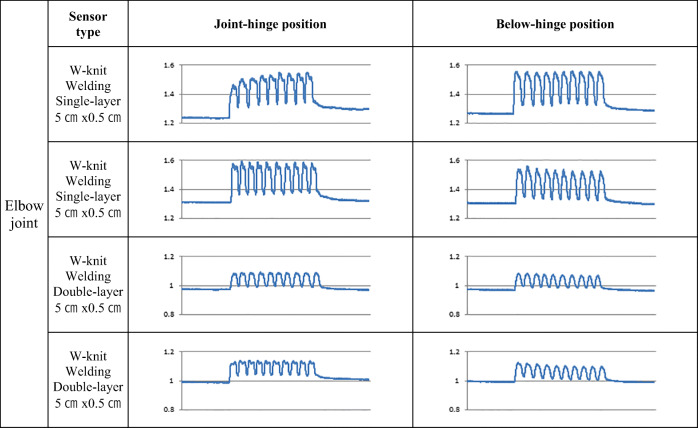
Electric resistance changes measured by CNT sensors during elbow joint attachment location test. (Y axis: resistance (Ω); X axis: repeats of the motion)

Double peaks were observed in the joint-hinge position of layer types, but relatively fewer double-peak phenomena were observed in the below-hinge position (Table 5).

#### Experiment 2-B

##### The CNT sensor and wearable platform

**Fig. 10:**
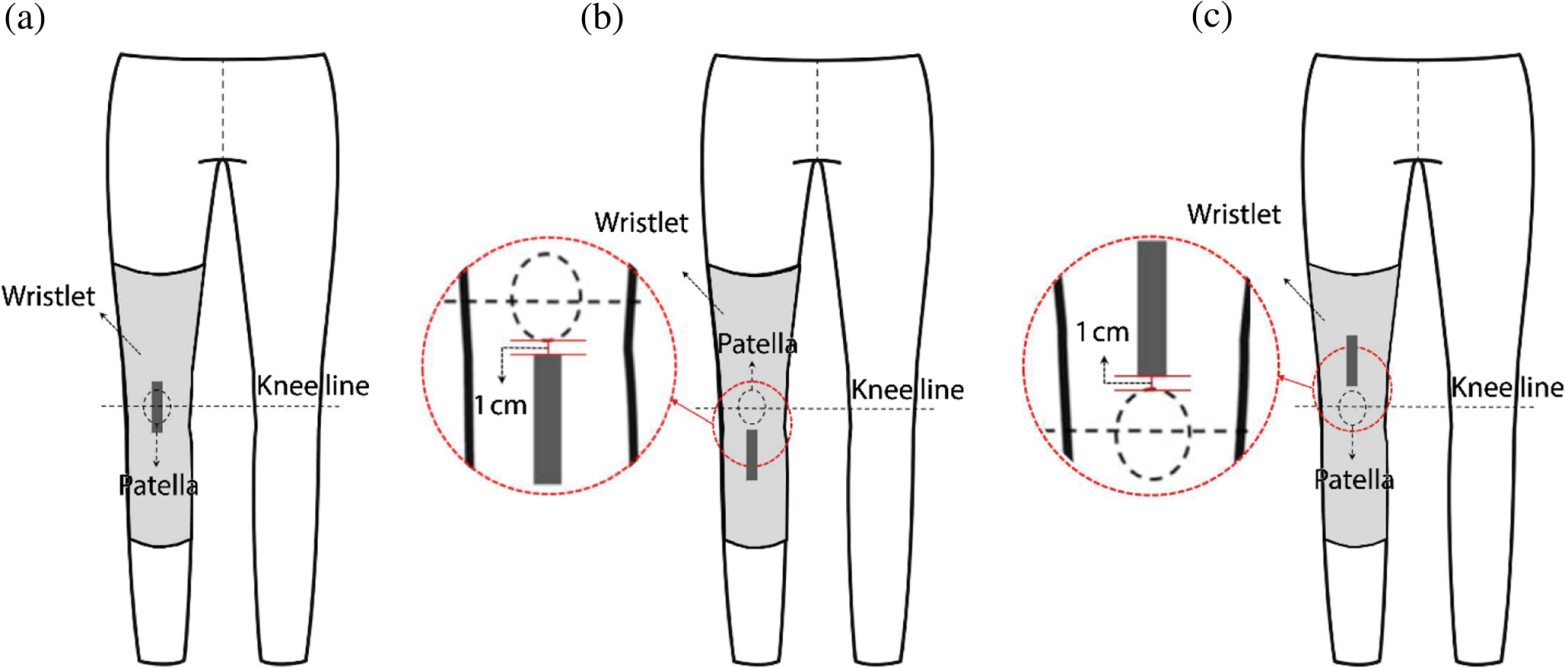
Schematic diagram of CNT sensor location attached around the patella. (**a**) Joint-hinge position; (**b**) below-hinge position; (**c**) upper-hinge position

The four types of CNT sensors were placed in the following positions around the knee joint: on the patella (knee hinge joint), called the “joint-hinge position,” below the patella, called the “below-hinge position,” and above the patella, called the “upper-hinge position” (Fig. 10). The sensor was stretched non-uniformly by each individual in motion due to each subject’s differing somatotype and distinct knee joint structure. Two points, 1 cm below the patella and 1 cm above the patella, were selected as determining the sensor’s mounting position. Sensor ends were placed 1 cm below and 1 cm above the patella to stably affix the interconnection part located at the end of the sensor.

In the matter of experimental design, a total of twelve sensor conditions (=4 × 3) on the wearable platforms were set up using a combination of four different sensor types (research variable #1) and three distinct attachment locations (the joint-hinge, below-hinge, and upper-hinge positions; research variable #2) around the knee.

**Fig. 11:**
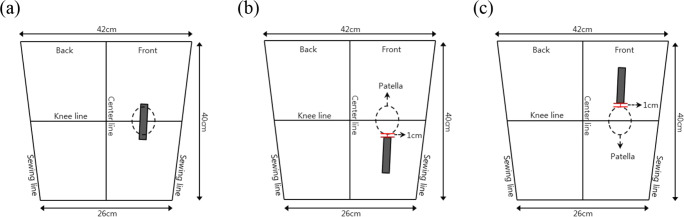
Schematic diagram of wristlet used for knee-joint CNT sensor location test. (**a**) Joint-hinge position; (**b**) below-hinge position; (**c**) upper-hinge position

CNT sensors were located vertically around the patella position transferred to a wristlet. The knee wristlets, a wearable platform were made based on the medium size of thigh circumference of Korean male to perform the test for CNT sensor attachment location (Fig. 11). All the materials for the fabrication of the wristlet were same as in Experiments 1 and 2-A.

##### Experimental setup

**Fig. 12:**
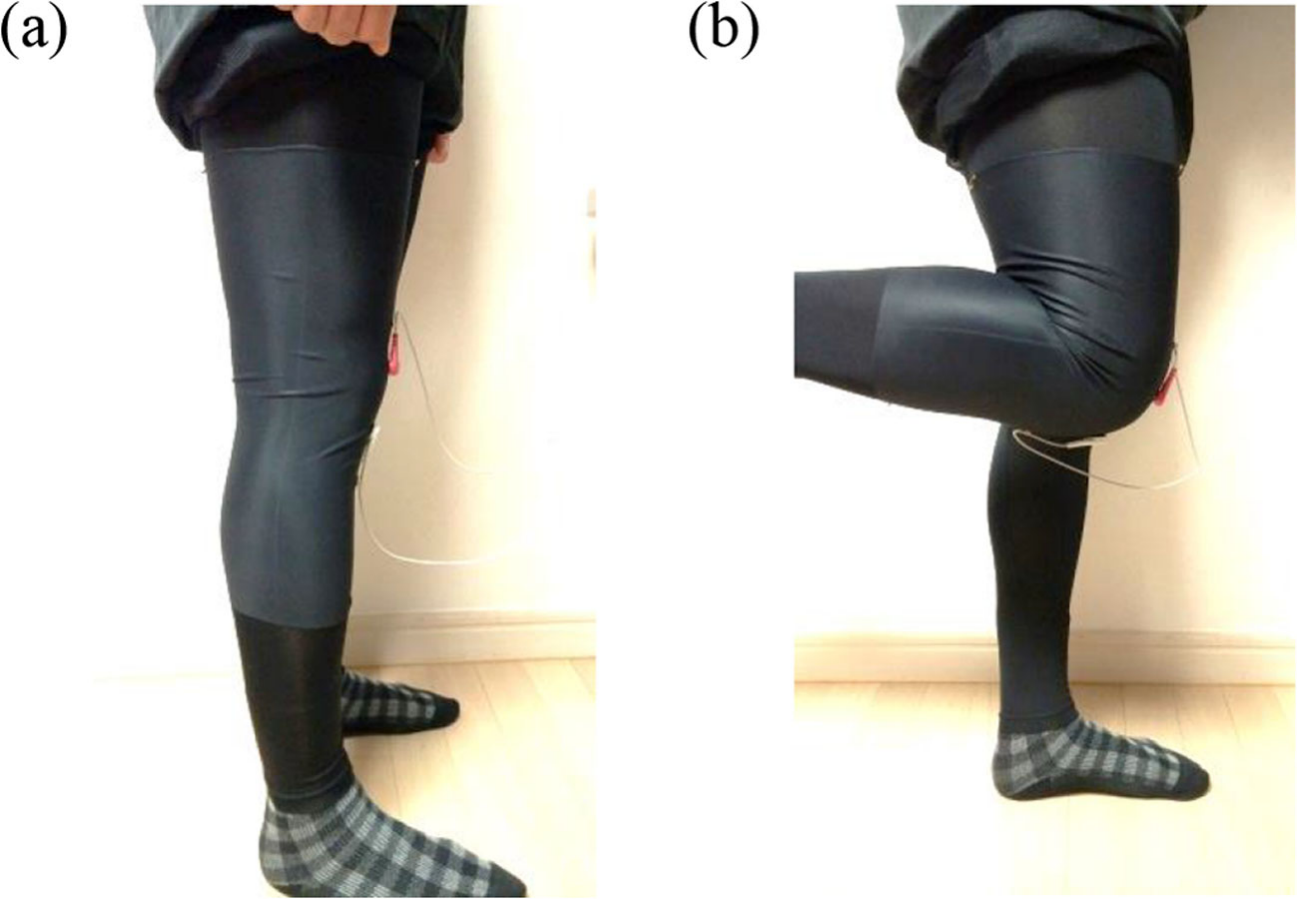
Test of knee-joint CNT sensor location in human subjects. Images of (**a**) uncontracted knee joint and (**b**) contracted knee joint

Each subject wore stretchy sports pants with a CNT sensor in the form of a wristlet attached to the right knee. Both ends of the wristlet were fixed to the sports pants by sewing to minimize slipperiness. Starting from an uncontracted knee in a standing position, one motion unit was defined as one complete flexion and relaxation of the right knee joint (Fig. 12). Each case was composed of 10 repetitive motions, and electric resistance changes were measured using an oscilloscope. The speed of one motion was set using a metronome as 0.5 Hz, which is understood to be a moderate exercise speed. Motion angle was determined by having individuals bend backward as far as possible and stretch their legs (around 90° - 110°). This angle varied depending on the leg thickness of each subject.

## Experimental results

Electric resistance changes were measured while subjects performed joint motion wearing a knee wristlet equipped with four types of CNT sensors. The results were analyzed using the same two criteria considered for Experiment 2-A.

Table 6 shows the average resistance obtained from five human subjects by repeating 10 knee joint motions. The first row in Table 6 shows the average resistance when the knees were relaxed and the second row shows the peak values when the knees were flexed. The third row shows the average resistance when the knees were relaxed again after one motion unit of relaxing and flexing. The values in the final row represent the differences in resistance in the relaxed state before and after the motion. The values in the shaded boxes indicate when the resistance changes were less than 0.005 Ω after the motion, implying that the baseline was stably reserved across repeats. More cases of baseline lift higher than 0.005 Ω were observed in general for single-layer cases, although this lift was decreased in sensors of 0.5 cm width set at the upper-hinge position and those of 1 cm width set at the below-hinge position. In double-layered sensors, more consistent and stable readings were obtained in all positions (Table 6). With regard to double-peak appearance, double-layer sensors showed reduced double-peak activity compared to single-layer sensors, as shown in Table 7. Overall, this result implies that the number of sensor layers has a more significant effect on measurement quality than sensor location does.

**Table 6:** Repetitive measurement averages in knee joint motion (Ω)

Form factor Position Motion	1 layer 5cm×0.5cm Joint	1 layer 5cm× 0.5cm Below	1 layer 5cm×0.5cm Upper	1 layer 5cm× 1cm Joint	1 layer 5cm× 1cm Below	1 layer 5cm× 1cm Upper	2 layers 5cm×0.5cm Joint	2 layers 5cm× 0.5cm Below	2 layers 5cm×0.5cm Upper	2 layers 5cm× 1cm Joint	2 layers 5cm× 1cm Below	2 layers 5cm× 1cm Upper
Relaxed	1.774	1.677	1.763	1.306	1.343	1.372	1.305	1.260	1.300	0.702	0.676	0.701
Flexed	2.022	1.985	2.010	1.597	1.547	1.568	1.440	1.414	1.436	0.778	0.768	0.779
Relaxed	1.781	1.687	1.766	1.314	1.348	1.378	1.306	1.262	1.300	0.704	0.678	0.701
Averaged baseline lift	0.007	0.010	0.003	0.008	0.005	0.006	0.001	0.002	0.000	0.002	0.002	0.000

(Shaded: Baseline lift resistance change after one unit motion ≤0.005 Ω; joint: joint-hinge position; below: below-hinge position; upper: upper-hinge position)

**Table 7:**
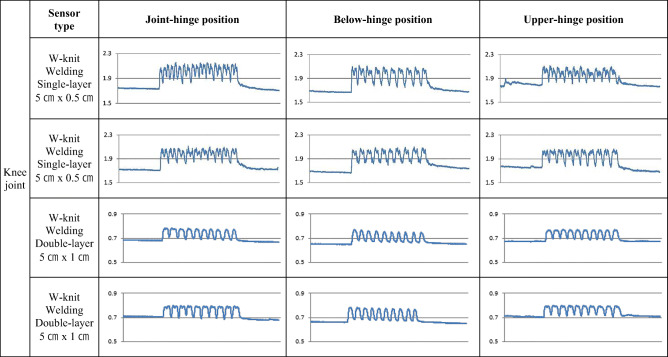
Electric resistance changes of CNT sensors from knee joint attachment location test. (Y axis: resistance (Ω); X axis: repeats of the motion)

Among the signals received from the double-layer sensor types in Table 7, those in the “below-hinge position” and “upper-hinge position” tended to exhibit reduced double-peak activity compared with the “joint-hinge position.” This observation likely implies that sensor location plays an ancillary role in determining measurement quality, although this only appears to be the case with double-layer sensor types.

## Discussion and conclusion

Our study identified the conditions for the CNT-based textile motion sensor, which can measure the joint motion in an efficient manner. In the first experiment, 48 types of CNT knit-based joint motion sensors were designed in order to monitor the joint movements of a dummy limb. The results of this preliminary experiment indicted that the most effective CNT sensors— those which showed the more stable and consistent resistance changes—were those with a base textile composed of 77% Polyester and 23% Polyurethane (W-knit), fixed on clothing via the welding method, in either single- or double-layers, with a sensor length of 5 cm, and with sensor widths of 0.5 cm or 1 cm. A total of four sensor types, including 5 cm × 0.5 cm and 5 cm × 1 cm (lengths x widths) sensors in both single- and double-layers, we used for further human subject experiments in Experiment 2.

In Experiment 2, it was observed that double-layer sensors maintained more stable baselines when detecting repetitive human joint motions than did single-layer structures. In addition, double-layer sensors showed reduced double-peak activity when sensors were placed at either the below-hinge or the upper-hinge position than they did when sensors were placed in the joint-hinge position. Based on these results, we find that using two sensors with a polyester-polyurethane mixed-knit, the welding method, double-layers, and dimensions of 5 cm × 0.5 cm and 5 cm × 1 cm of length x width is the best way to ensure effective limb motion sensing. This offers insight into an important indicator for the development of motion-sensing sportswear. However, it should be noted that motions sensing sportswear needs to be researched for various speeds of sports activity, as our research result is limited to the slow limb motion (0.5 Hz) similar to the speed of typical weight training.
